# A self-reported Brazilian registry of 5q-spinal muscular atrophy: data on natural history, genetic characteristics, and multidisciplinary care

**DOI:** 10.1055/s-0044-1792096

**Published:** 2024-12-20

**Authors:** Rodrigo Holanda Mendonça, Juliane Suellen Arndt de Godoi, Edmar Zanoteli

**Affiliations:** 1 Universidade de São Paulo, Faculdade de Medicina, Departamento de Neurologia, São Paulo SP, Brazil.; 2 Instituto Nacional da Atrofia Muscular Espinhal, São Paulo SP, Brazil.

**Keywords:** Muscular Atrophy, Spinal, Survival of Motor Neuron 1 Protein, Natural History, Registries, Atrofia Muscular Espinal, Proteína 1 de Sobrevivência do Neurônio Motor, História Natural, Sistema de Registros

## Abstract

**Background** Spinal muscular atrophy linked to chromosome 5q (SMA-5q) is a neurodegenerative disorder caused by mutations in the *SMN1* gene.

**Objective** To describe the key demographic, clinical and genetic characteristics, as well as natural history data of patients with SMA-5q.

**Methods** Up to January 2022, 706 patients with confirmed genetic diagnosis of SMA-5q, or their parents, completed a self-reported questionnaire on natural history, genetic characteristics, drug treatments, and multidisciplinary care.

**Results** Most patients had type 1 SMA-5q (42%); with 33% having type 2, and 23% type 3. There were 667 patients (94.4%) with a homozygous *SMN1* -exon 7 deletion. Of the total, 131 (18.6%) patients had a previous family history of the disease, and the familial recurrence rate was higher in type 3 (25.6%). Type 1 patients had a mean age of 3 months at the onset of symptoms and a delay of more than 3 months until genetic diagnosis. The median survival of patients with type 1 without invasive ventilation was 27 months. Before 2018, the median age of use of invasive ventilation was 16 months and, after, most patients (71%) were not submitted to invasive ventilation. About 50% of patients with type 3 lost their walking ability by 37 years of age. Further, 384 (54.4%) patients had access to disease-modifying therapy, and 62.3% of type 1 patients were in treatment, compared with only 47.2% of type 2 and 31.9% of type 3 patients.

**Conclusion** There is still a substantial diagnostic delay, especially in those patients with types 2 and 3 SMA-5q. However, the present study demonstrated prolonged survival, especially in type 1 patients.

## Resumo

**Antecedentes** A atrofia muscular espinhal ligada ao cromossomo 5q (AME-5q) é uma doença neurodegenerativa causada por mutações no gene *SMN1* .

**Objetivo** Descrever as principais características demográficas, clínicas e genéticas, assim como dados de história natural de pacientes com AME-5q no nosso meio.

**Métodos** Até janeiro de 2022, 706 pacientes com diagnóstico genético confirmado de AME-5q, ou seus pais, preencheram questionário sobre dados de história natural, características genéticas, tratamento medicamentoso e cuidados multidisciplinares.

**Resultados** A maioria dos pacientes tinha AME-5q tipo 1 (42%); 33% tinham tipo 2 e 23% tipo 3. Deleção homozigótica no *SMN1* foi notada em 667 pacientes (94,4%). Do total, 131 (18,6%) pacientes tinham história familiar prévia, e a taxa de recorrência familiar foi maior no tipo 3 (25,6%). Os pacientes com tipo 1 tinham idade média de 3 meses no início dos sintomas e atraso de mais de 3 meses até o diagnóstico genético. A sobrevida mediana de pacientes com tipo 1 sem ventilação invasiva foi de 27 meses. Antes de 2018, a idade mediana de uso de ventilação invasiva era de 16 meses e, após, a maioria dos pacientes (71%) não foi submetida a ventilação invasiva. Cerca de 50% dos pacientes com tipo 3 perderam a marcha em média aos 37 anos de idade. Além disso, 384 (54,4%) pacientes tiveram acesso a alguma terapia modificadora da doença; 62,3% dos pacientes tipo 1 estavam sendo tratados, comparados a 47.2% do tipo 2 e 31.9% do tipo 3.

**Conclusão** Ainda existe um atraso substancial para o diagnóstico, especialmente nos pacientes com AME-5q tipos 2 e 3. Contudo, o presente estudo demonstrou sobrevida prolongada em pacientes tipo 1.

## INTRODUCTION

Spinal muscular atrophy (SMA) is a neurodegenerative disorder of lower motor neurons from the spinal cord and motor nuclei of the brainstem, leading to severe proximal and symmetrical weakness associated with ventilatory insufficiency and spinal deformity. 1 The most prevalent form is caused by deletions or disease-causing variants in the survival motor neuron 1 ( *SMN1* ) gene, located in chromosome 5q (SMA-5q), which segregates as an autosomal recessive trait. 2

The classification of SMA-5q in at least three subtypes depends on the age of disease onset and the achievement of motor milestones. 1 Type 1 SMA-5q is a severe form characterized by onset at 0 to 6 months, and children cannot sit unaided. 3 In type 2 SMA-5q, the clinical manifestations start between 6 and 18 months of age, and children cannot walk unaided. Children with type 3 SMA-5q manifest the disease in the second year of life or later and can walk unaided. 1 Many patients present intermediate phenotypes between types 1 and 2. 4 Outliers of this classification have been recognized, including a mild adult form, or type 4, with slow progression of weakness. 3 4

The incidence of SMA-5q is around 1 every 10,000 live births. 5 Unfortunately, we do not have previous Brazilian data on the incidence and prevalence of the disease. The Brazilian National Institute of Spinal Muscular Atrophy (Instituto Nacional da Atrofia Muscular Espinhal, INAME, in Portuguese) is a nongovernmental association of patients and families with the disease. It aims to promote public policies to improve care for patients and health professionals' knowledge of the disease and its clinical care. The institute has an extensive Brazilian patient database, in which approximately 2,000 registered patients received a presumed diagnosis of SMA-5q.

The present study aimed to describe the key demographic, clinical and genetic characteristics, and natural history data of patients with SMA-5q in Brazil through a self-reported registry from patients who are in the INAME database.

## METHODS

The INAME registry aims to obtain retrospective and longitudinal data from Brazilian patients with SMA-5q, and here, we report on the initial retrospective phase. Patients or families from the INAME database were invited to participate in the self-reported registration. The project was approved by the ethics committee of the Instituto Dante Pazzanese de Cardiologia (#38534320.6.0000.5462), and the patients who agreed to participate in the study signed an informed consent form electronically.

This registry was based on the Registries Core Dataset (TREAT-NMD SMA, Newcastle, England). 6 Patients or family members answered 117 questions, subdivided into seven sections, encompassing various aspects including demographic data, socioeconomic profile, diagnosis (time of symptoms onset, time to diagnosis, genetic characteristics), drug treatment (start of disease-modifying drugs, switch), clinical observations (comorbidities, hospitalization, loss of motor functions, time to ventilatory support), multidisciplinary interventions (surgeries, respiratory and motor physiotherapy, nutritional support and speech therapist, motor scales evaluation), and health service providers (health insurance, public health system, home care). Additionally, the data collected in the online questionnaire utilizing Google Forms (Google LLC., Mountain View, CA, USA) was transferred to a digital database in an Excel (Microsoft Corp., Redmond, WA, USA) file for further analysis.

Data were collected from January 2021 to 2022. Inclusion criteria were patients with a confirmed molecular diagnosis of SMA-5q (regardless of age), and patients were asked to upload the molecular test to the system. The neurologists of the scientific committee (EZ and RHM), who specialized in neuromuscular diseases, reviewed all genetic tests; patients without a molecular diagnosis of SMA-5q were excluded. We rely on the diagnosis provided by the patient's doctor. Additionally, the scientific committee reviewed the agreement between the type of SMA and maximum motor milestone provided. On several occasions, the study's auxiliary team contacted patients or family members directly (by telephone) to help complete the questionnaire or collect missing data.

### Statistical analysis

Quantitative variables were described by median and interquartile range (IQR), while absolute and relative frequencies described qualitative variables. The Kruskal-Wallis test was used to compare quantitative variables between types of SMA. For time-to- event outcomes, the Kaplan-Meier estimator was used for survival curves, and the log-rank test was used to compare groups. All analyses were performed using the R (R Foundation for Statistical Computing, Vienna, Austria) software, and we consistently considered a *p* -value less than 0.05 indicative of statistical significance.

## RESULTS

Among the 900 patients in the INAME database who were successfully contacted, 750 completed the online survey. Of these, 44 were excluded because they reported either not having a molecular diagnosis or not uploading the test to the system. Thus, we included 706 patients in this analysis.

### Demographic data

**Figure 1:**
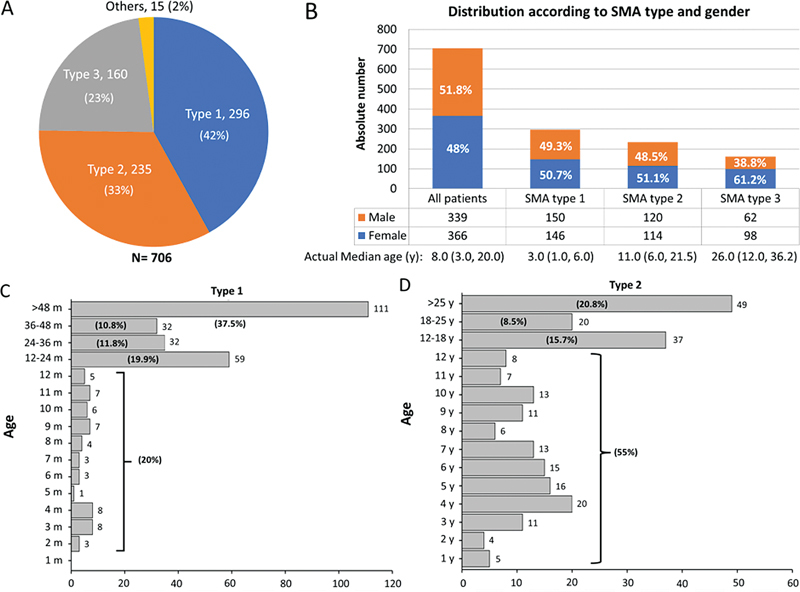
( **A** ) The distribution of SMA-5q types in the registry. ( **B** ) Gender distribution in the general group, and according to types and median age in each subtype. ( **C** ) The age distribution in type 1. ( **D** ) The age distribution in type 2.

The most prevalent subtype was SMA-5q type 1 (44%). Among the others, 33% had type 2, and 23% had type 3. There were seven patients with type 0 SMA-5q, and eight had type 4 ( Figure 1A ). Most patients were from the Southeastern region (51%) of Brazil, while others were from the Northeastern (19.5%), Southern (16.9%), Midwestern (7.6%) and Northern (5%) regions. Distribution according to sex was similar in the total study population and in those with types 1 and 2. Among patients with the milder form, there was a slight predominance of females (61.2%, Figure 1B ).

The median age of patients with type 1 SMA-5q was 3.0 (1.0–6.0) years ( Figure 1C ). At the time of data cut-off, 37.5% of patients with type 1 were older than 4 years. The median age of patients with type 2 was 11.0 (6.0–21.5) years, and 45% were adolescents or adults ( Figure 1D ). The median age of patients with type 3 was 26.0 years (12.0–36.2).

### Diagnosis and genetic data

Regarding mutations in the *SMN1* gene, 667 patients (94.4%) had a homozygous *SMN1* -exon 7 deletion. There were 38 patients (5.5%) with a heterozygous deletion in *SMN1* and a point mutation in the other allele. Only one patient had a point mutation in both *SMN1* alleles (p.Gly261.Leufs*8); in this situation, the parents were consanguineous. The molecular test used for diagnosis in 515 (73%) patients was multiplex ligantion-dependent probe amplification (MLPA). The polymerase chain reaction (PCR) for detecting homozygous *SMN1* -exon 7 deletion was used in another 191 (27%).

**Figure 2:**
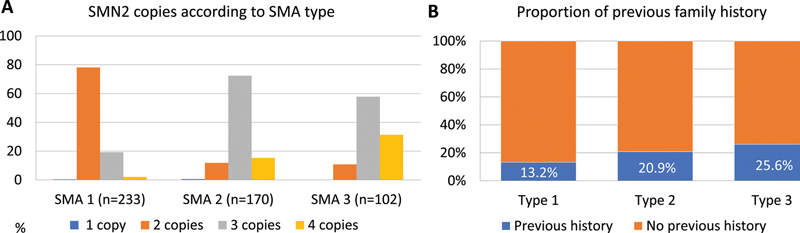
( **A** ) The distribution of *SMN2* copy number according to SMA-5q types in those with MLPA confirmation ( *n* = 505, types 0 and 4 not included). ( **B** ) The proportion of previous family history of SMA-5q according to patients' type. Note a more significant proportion of recurrence cases in type 3.

Regarding the *SMN2* copy number, two copies of *SMN2* occurred in 78.1% of SMA-5q type 1 patients. Most patients with type 2 (72.4%) presented three copies of *SMN2* , and 31.4% of type 3 presented four or more copies ( Figure 2A ). From the total, 131 (18.6%) patients had a family history of the disease, and the familial recurrence rate was higher in those with type 3 (25.6%), as shown in Figure 2B . The consanguinity rate was low, and only 37 (5.2%) patients noted some degree of relation between the parents.

Regarding the time until diagnosis, patients with type 1 had a mean age of 3 months at the onset of symptoms and an additional 3 months elapsed until genetic diagnosis ( Table 1 ). In 2018, the pharmaceutical industry began offering free molecular testing (MLPA and gene sequencing). Thus, 55% of the diagnostic tests registered in this study were performed in 2018. From that year on, the average time for genetic diagnosis of all types of SMA-5q shortened from 14 to 9 months. Awareness about the signs and symptoms of the disease also increased, which shortened the time for clinical diagnosis of type 2 and 3 from 11 to 9 months and from 24 to 22 months, respectively ( Table 1 ).

**Table 1:** Time elapsed in the natural history of the disease from symptom onset to diagnosis and initiation of treatment, and the difference in diagnostic times before and after 2018

Clinical characteristics	Total N = 706	SMA1 N = 296	SMA2 N = 235	SMA3 N = 160	*p* -value a
Time between birth and symptom onset (mo) (median, IQR)	7.0 (2.0–18.0)	3.0 (1.0, 5.0)	10.0 (6.0–15.0)	27.0 (15.0–50.5)	< 0.001
Time between symptom onset and clinical diagnosis (mo) (median, IQR)	6.0 (1.0–18.0)	2.0 (1.0, 4.0)	10.0 (4.0–20.0)	24.0 (9.0–89.0)	< 0.001
Time between symptom onset and genetic diagnosis (mo) (median, IQR)	14.0 (3.0–95.5)	3.0 (2.0, 6.0)	29.0 (11.5–181.5)	169.5 (25.2–296.2)	< 0.001
Time between clinical diagnosis and initiation of DMT (mo) (median, IQR)	14.0 (3.0–95.5)	6.0 (2.0–29.0)	37.5 (12.0–116)	*	< 0.001
**Time between symptom onset and clinical diagnosis (mo) (median, IQR)**					
Before 2018	8.0 (2.0–23.0)	2.0 (0.0–4.0)	11.0 (5.8–24.0)	24.0 (6.2–89.0)	< 0.001
After 2018	3.5 (1.0–14.0)	2.0 (0.0–4.0)	9.0 (3.0–18.0)	22.0 (11.2–53.0)	< 0.001
**Time between symptom onset and genetic diagnosis (mo) (,median, IQR)**					
Before 2018	18.0 (5.0–84.0)	4.0 (2.0–9.8)	53.5 (14.2–227.8)	164.0 (24.5–311.0)	< 0.001
After 2018	10.0 (2.0–100.5)	3.0 (1.0–5.0)	18.0 (7.0–43.5)	106.5 (25.2–255.5)	< 0.001

Abbreviations: DMT, disease-modifying therapy; IQR, interquartile range; mo, months. Notes: ^a^ Kruskal-Wallis test, *data unreliable because most SMA type 3 patients did not have access to therapies.

### Natural history data

**Figure 3:**
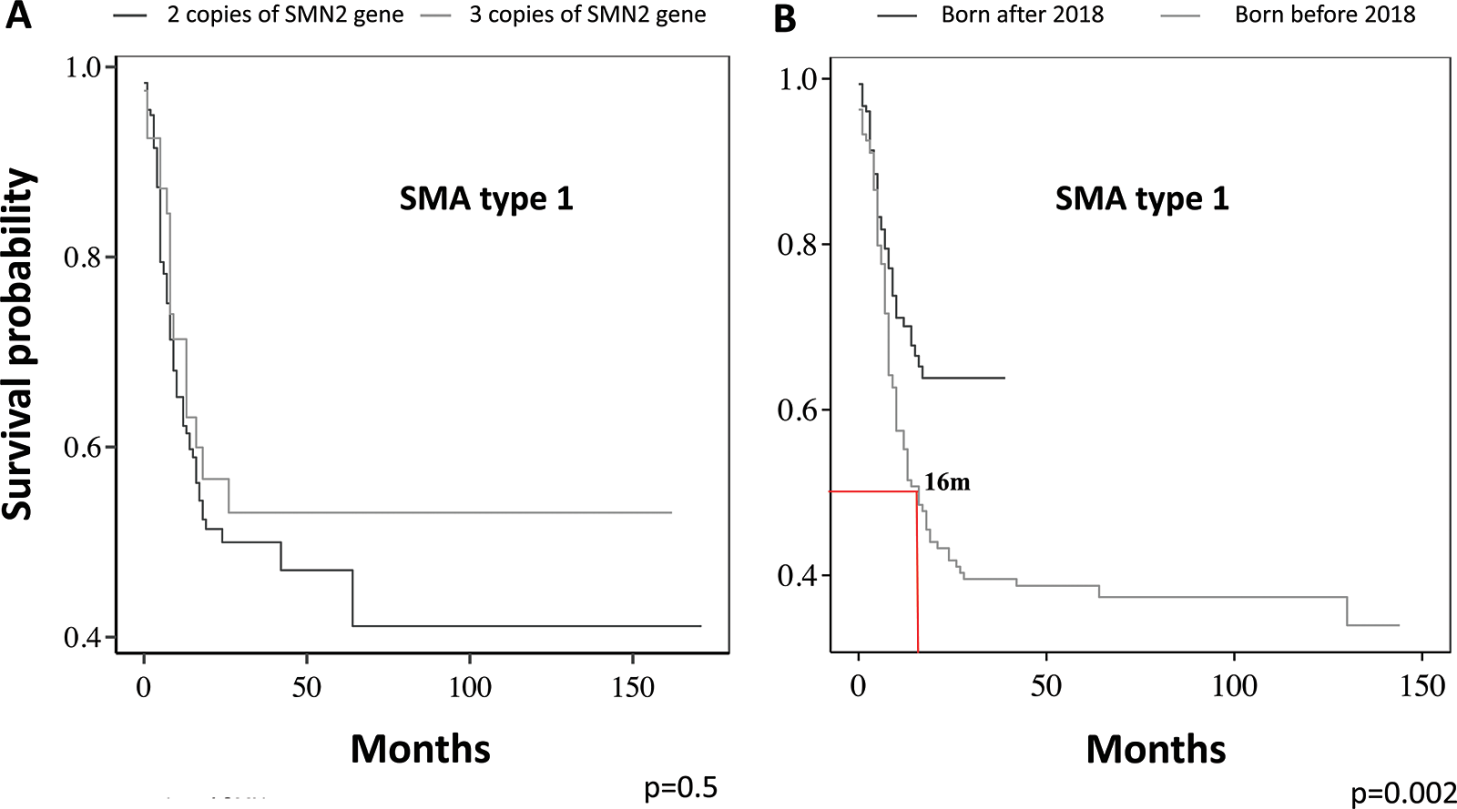
( **A** ) The Kaplan-Meier curve for invasive ventilation-free survival in patients with type 1 according to *SMN2* copy number. For patients with two copies of *SMN2* , the median time was 24 months and did not differ significantly from those with three copies ( *p* = 0.5). ( **B** ) The survival curve relative to the time until invasive ventilation of type 1 patients, according to birth year. In those born before 2018, the median time until invasive ventilation use was 16 months (IQR: 10–27), and for those born after 2018, it was not possible to estimate because less than 25% of patients achieved this outcome ( *p* = 0.002).

In type 1 SMA-5q patients, 50% underwent invasive ventilation up to 27 months of age. For patients with two copies of *SMN2* , the median time was 24 months. It was impossible to estimate the median in those patients with three copies, although no difference was observed between the survival curves ( *p* = 0.5), as shown in Figure 3A . The survival curve relative to the time until invasive ventilation in type 1 patients, according to birth year, shifted starting in 2018. For those born before 2018 and, as such, did not receive treatment with disease-modifying drugs, the median time until the use of invasive ventilation was 16 (IQR: 10–27) months, and for those born after 2018, the median could not be estimated because only 29% of patients underwent invasive ventilation ( *p* = 0.002, Figure 3B ).

**Figure 4:**
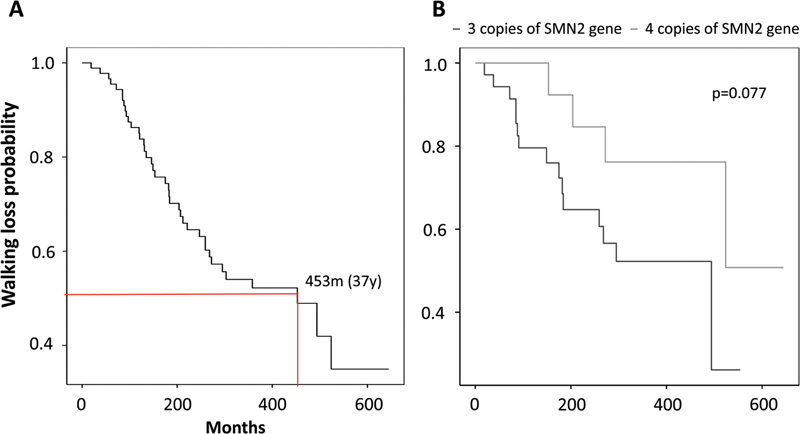
( **A** ) The estimated curve of walking loss in SMA-5q type 3. About 50% of type 3 patients lost walking ability by 453 months (37 years) of age. ( **B** ) The Kaplan-Meier curve of gait loss in type 3 according to *SMN2* copies. The outcome appeared to be earlier in those patients with three copies of *SMN2* , a median of 36 years (first quartile of 15 years) compared with those with four copies, with a *p* -value close to statistical significance ( *p* = 0.077).

Other natural history data obtained included the loss of gait in type 3 SMA-5q patients. Patients answered the date on which they lost the ability to walk for at least 10 m without support. The median of walking loss was 37 years, with a first quartile of 22.3 years ( Figure 4A ). Gait loss appeared earlier in patients with three copies of *SMN2* than in those with four. The Kaplan-Meier curve of gait loss according to *SMN2* copy number showed a *p* -value close to statistical significance ( *p* = 0.077, Figure 4B ). The average age of lost independent walking ability was 12 (IQR: 8–21) years.

### Multidisciplinary care and previous surgery

**Figure 5:**
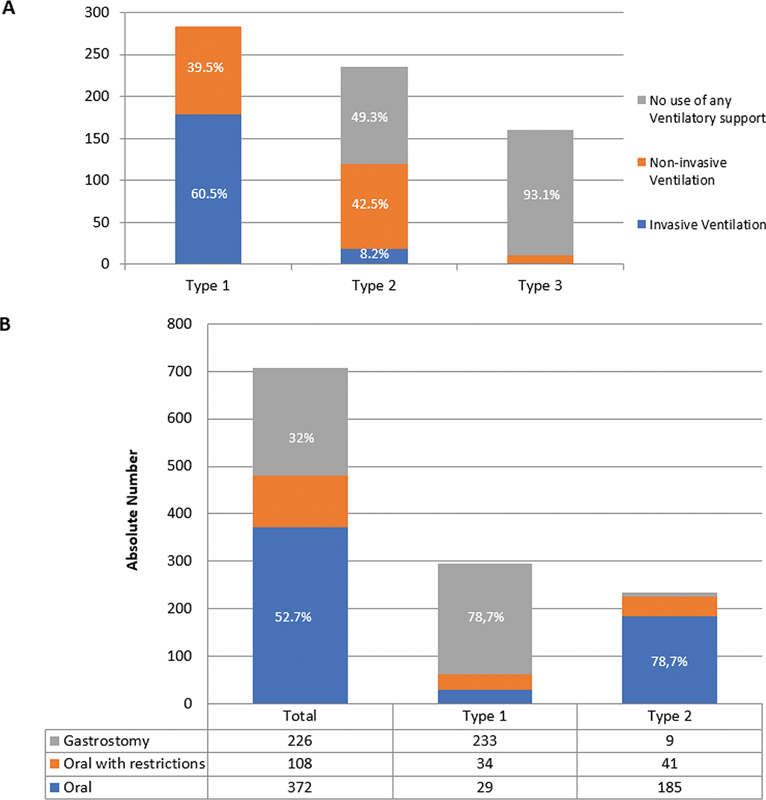
( **A** ) The distribution of ventilatory support according to SMA-5q types. ( **B** ) The level of nutritional support/feeding route according to types.

As mentioned before, most patients with type 1 SMA-5q (60.5%) were on invasive ventilation. Among type 2 patients, 49.3% did not use any ventilatory support, and 42.5% used noninvasive ventilation for some period ( Figure 5A ). Regarding respiratory assistance, 411 (58.2%) patients received respiratory physiotherapy at least once weekly. There were 200 patients (28.3%) who said they knew or performed the air stacking technique with a bag valve mask. Although many reported undergoing respiratory physiotherapy, only 276 (39.1%) had already performed a vital capacity test or spirometry. Only 33% said they knew about or used cough-assist machines.

Regarding motor rehabilitation, 534 patients (75.6%) said they underwent motor physical therapy at least once a week. Of these, 359 (50.8%) underwent it more than twice a week. Despite this, only 379 (53.7%) responded that they had already been evaluated through some motor function scale. The proportion of patients evaluated by motor scales according to the SMA-5q types did not differ between groups, with 175 (59.1%) in type 1 and 118 in types 2 (50.2%) and 3 (52.5%).

Gastrostomy was used by 32% of patients ( Figure 5B ). The use of gastrostomy was higher in type 1 (78.7%) patients, with an inversion of this proportion occurring in type 2, with 21.3% needing a gastrostomy tube. Additionally, 299 (42.3%) patients said they had access to a speech therapy professional at least once a week. Scoliosis was reported in 54.4% of all patients and was higher in type 2 patients (70.6%), followed by those with type 3 (57.5%), and type 1 (42.2%). A total of 21% patients underwent scoliosis surgery, more than half of whom had type 2.

At least 101 patients (14.3%) reported one unplanned hospitalization last year. Patients with type 1 SMA-5q reported having had one or two unplanned hospitalizations in the previous year (24%), compared with the other types of SMA-5q (type 2: 6.3%, type 3: 4.3%, *p* < 0.001).

### Disease-modifying therapies (DMTs)

By the cut-off date in January 2022, 384 (54.4%) patients had access to some DMTs. Most patients (359; 93.5%) were using nusinersen, as it was the only drug available through the Brazilian public health system. Another 18 (4.7%) patients reported using gene therapy, and 7 (1.8%) used risdiplam. The median treatment duration was 15 (IQR: 6–26) months.

When stratifying access to nusinersen use according to SMA-5q types, 62.3% of patients with type 1, 47.2% of type 2, and 31.9% of type 3 patients were using this drug in treatment. This difference is justified because, at the time of the data analysis, nusinersen was only distributed in the public health system for patients with type 1 SMA-5q without permanent invasive ventilation use.

## DISCUSSION

The present study is the first SMA-5q patient registry in Latin America. 7 Although subject to systematic errors and lack of information, self-reported registries by patients with neuromuscular diseases have proven to be useful and provide realistic results. 8 9 10 11 The self-reported data here are comparable to other registries, and the proportion between the SMA-5q types is similar to previous studies. 12 Although the data presented here is about prevalence and not incidence, 42% of the patients in our study reported having type 1, which may reflect an ascertainment bias of our database. Interestingly, a recently published Italian survey showed a prevalence of type 1 in only 22.6% of patients in the era of DMTs, the same context as our study. 13

Also corroborating the quality and reproducibility of our results is that the number of patients with a homozygous deletion in the *SMN1* gene was closer to that reported in previous studies, 14 at around 94.4% of patients. In a previous Brazilian study, authors reported less than 90% of SMA-5q patients with homozygous exon 7-SMN1 deletion, perhaps because they presented a higher proportion of patients with milder disease phenotypes. 15

The diagnostic journey of SMA-5q in our population still involves challenges. The mean age at onset of symptoms for type 1 was similar to other studies, ranging from 2 to 3 months old. 16 17 Considering the time from symptom onset to genetic diagnosis, a delay is seen in all types of SMA-5q. The diagnostic delay exhibited an inverse relationship with disease severity, with a median duration of 3 months for type 1, 29 months for type 2, and 169 months for type 3, reflecting longer diagnostic delays than those previously reported in systematic reviews, 16 especially in types 2 and 3. Although the time between symptom onset and clinical and genetic diagnosis has improved since 2018, there is still a gap, considering that the final diagnosis time for type 1 has only improved by 1 month since 2018. 17 Another gap in our study is the difficulty in accessing therapies within the country, which prevents us from determining treatment times correctly, starting from the symptom onset, especially in type 3 SMA-5q.

Regarding the genetic data of our population, the presence of consanguinity was low in this study's population (less than 5%), which shows considerable regional variability. A recent Iranian registry showed a rate of 52.4% of consanguinity. 18 The low rates in our population alerts us to a possible high prevalence of asymptomatic carriers for *SMN1* deletion, which should be in the order of 1 to 37 people, as reported by another study in the Brazilian population. 19 The high recurrence rate of type 3 SMA-5q (around 25%) was similar to the report by a recent Iranian registry. 18 Given the milder phenotype, it may reflect a longer diagnostic delay, preventing adequate genetic counseling.

It was possible to evaluate survival beyond the expected span for patients with SMA-5q type 1 because it was the most prevalent form in our registry (42%). Of those, 37.5% were older than 4 years, unlike the data reported by current SMA-5q registries. 13 18 This finding may be associated with an improvement in multidisciplinary care and the introduction of DMTs. 20 21 Additional information from this work is the large number of adolescent and adult patients in the sample, with 45% of type 2 patients being over 12-years-old and type 3 patients having a median age of 26. These data reflect the need to develop scales and surveys for this group concerning disease progression and quality of life. 22 23

Natural history data in the Brazilian population are unpublished, however, the mean age at the start of invasive ventilation in type 1 SMA-5q patients is already much better than that reported by other natural history studies. The patients in this registry had a median survival of 27 months until invasive, permanent ventilation, compared with 8 months in the Neuronext study, 24 and 13.5 months in the PNCR study. 3 However, evaluating ventilation-free survival before and after 2018, when DMTs started to be used (mainly nusinersen), we found a drastic change from a median of 16 months data like that of the PNCR study, 3 to an estimate that cannot be measured after 2018. This suggests the effectiveness of current therapies in modifying the natural history of type 1 SMA-5q together with an earlier diagnosis and introduction of multidisciplinary care.

Loss of walking ability in type 3 SMA-5q patients is also important natural history data. A recent observational study showed 14-years-old was the time of motor skills loss, compared with a median age of 12-years in our registry. 25 Currently, this is the only SMA-5q population in our country that does not have access to DMTs through the public and universal healthcare system, and progressively loses functionality and independence, which is characteristic of a disease that occurs at all ages. 26

This study has some limitations. First, it consisted of a survey answered by patients and their families, in which there may be interviewer and social desirability bias, mainly regarding the more technical data. Second, many answers about natural history depend on the memories of the interviewees regarding the dates or periods in which losses of motor functions occurred, which may lead to recall bias. Additionally, we lack the exact data regarding the age of permanent ventilation. Instead, we only have data on the age at which invasive ventilatory support was initiated. Finally, another limitation is the possibility that our registry does not faithfully represent the Brazilian population, since we do not know the actual adherence of patients in the INAME database.

As a future direction concerning access to DMTs, the data need to be updated, given the constant changes in local protocols making new therapies available, and including patients with type 3 SMA-5q as eligible for treatment in the public health system of Brazil. 27 28 29 The prospective phase is intended to correct this limitation, updating the data regarding access to therapies. In addition, the prospective phase is intended to better assess the incidence of intercurrences and hospitalizations and to evaluate improvements implemented in multidisciplinary care, in addition to better assessing the survival of the most severe forms with the institution of therapies.

In conclusion, the results of the present study are unprecedented in the Brazilian population, as this is the country's first SMA-5q registry. There is a substantial diagnostic delay in the Brazilian population, especially in those with types 2 and 3 SMA-5q, but there has been an improvement since 2018. Natural history data already demonstrate prolonged survival, especially for type 1 patients, and the outcome of invasive ventilation has changed dramatically since the advent of DMTs. Patients with type 3 SMA-5q suffer from progressive loss of gait and functionality and lack broad access to new therapies.
